# Supplementary material for: Scn1a gene reactivation after symptom onset rescues pathological phenotypes in a mouse model of Dravet syndrome
Source: Nat Commun. 2022 Jan 10;13:161. doi: 10.1038/s41467-021-27837-w (PMC8748984; doi:10.1038/s41467-021-27837-w)
Supplement: Supplementary file 1 — Supplementary Information [file 41467_2021_27837_MOESM1_ESM.pdf]

Supplementary Figure 1

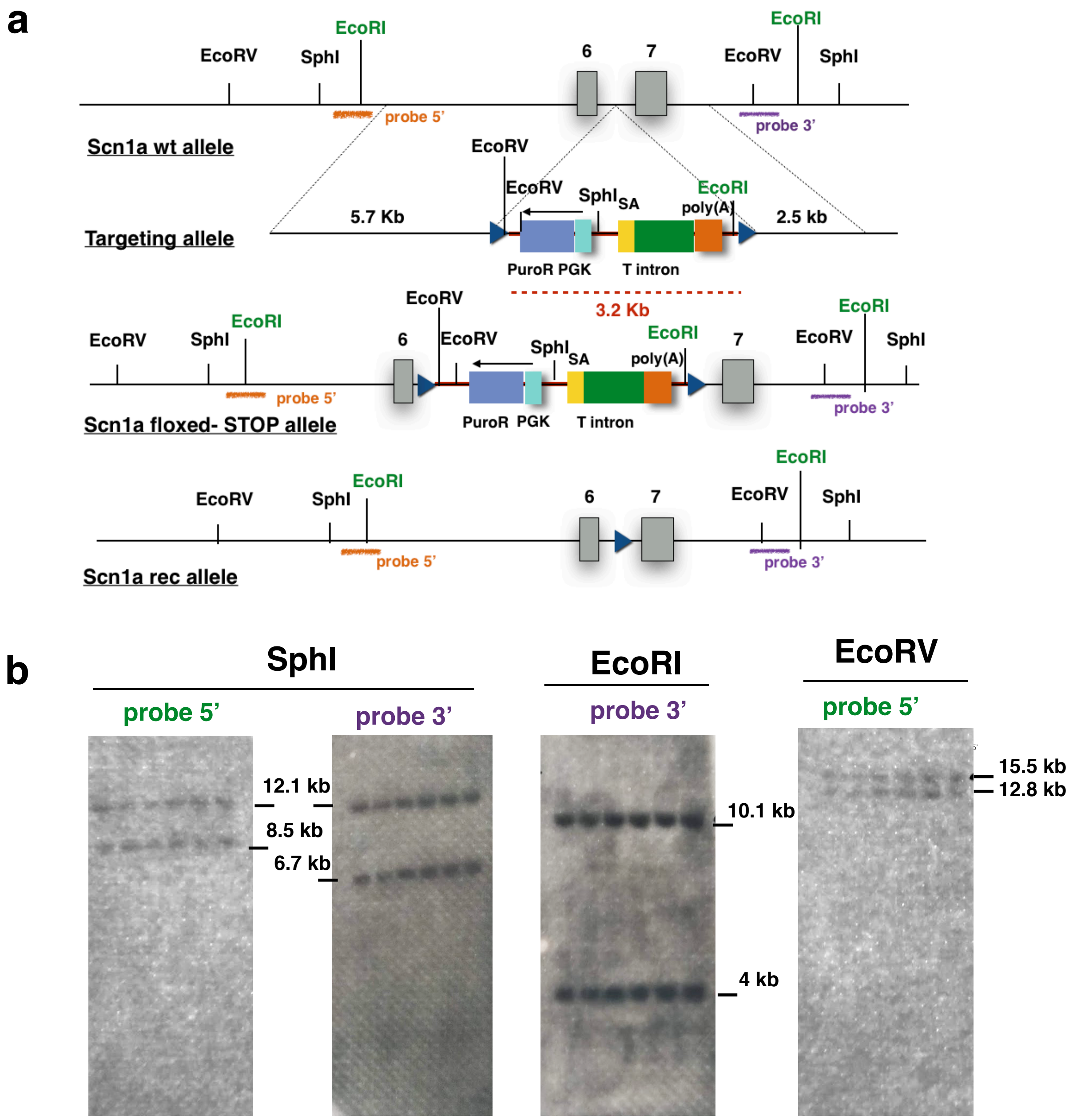

Supplementary Figure 1| Genetic strategy for the reversible DS model.

(a) Scheme of the gene targeting construct generated to insert in the intron between exon 6 and 7 of Scn1a gene a floxed STOP cassette composed by a splicing acceptor (SA) - a Puromycin resistance – a STOP codon (loxP-SA-PGK Prom-PuroR-STOP-loxP). **(b)** Southern blot of 6/103 clones in which heterozygous targeting occurred at *Scn1a* gene locus. Genomic DNA was digested with SphI, EcoRI or EcoRV and membranes were hybridized with probes tagging 5'arm or 3'arm. 2 independent experiments were performed, the first as a screening, the second to confirm positive clones.

Supplementary Figure 2

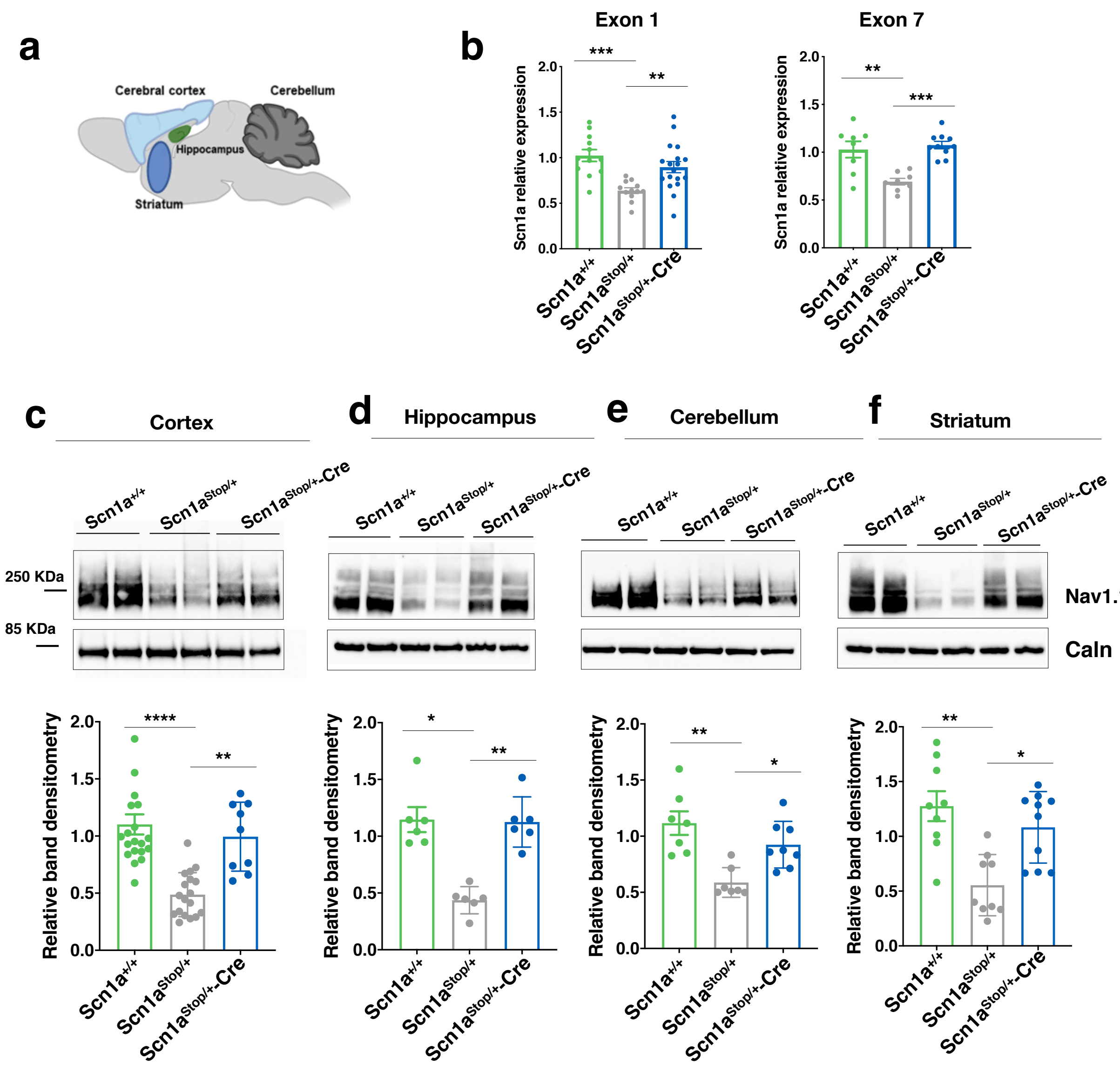

Supplementary Figure 2| Expression of *Scn1a* mRNA and Na_v1.1 protein in *Scn1a*$^{Stop/+}$ mice after PHP.eB-Cre injection at P30 (*Scn1a*$^{Stop/+}$-Cre).

(**a**) Schematics depicting brain regions dissected to extract RNA and proteins analyzed by qRT-PCR and WB, respectively. Created with BioRender.com. (**b**) Results from qRT-PCR showing *Scn1a* mRNA levels in *Scn1a*$^{+/+}$, *Scn1a*$^{Stop/+}$, and *Scn1a*$^{Stop/+}$ -Cre mice with primers tagging exon 1 (**p=0.0081;***p=0.0003, n=12 cortical hemispheres over 6 independent brains for *Scn1a*$^{+/+}$, n=12 cortical hemispheres over 6 independent brains for *Scn1a*$^{Stop/+}$, and n=18 cortical hemispheres over 9 independent brains for *Scn1a*$^{Stop/+}$ -Cre) and exon 7 (**p=0.0012; ***p=0.002, one-way ANOVA followed by Tukey's multiple comparison, n=8 cortical hemispheres over 4 independent brains for *Scn1a*$^{+/+}$ n=8 cortical hemispheres over 4 independent brains for *Scn1a*$^{Stop/+}$, and n=10 cortical hemispheres over 5 independent brains for *Scn1a*$^{Stop/+}$ -Cre). (**c-f**) Representative WB for Na_v1.1 and Calnexin protein obtained from membrane-enriched protein extracts (*up*) and densitometric quantification of immunoreactive Na_v1.1 bands normalized on calnexin (*down*) are presented (cerebral cortex, **** p<0.0001, n=20 cortical hemispheres over 10 independent brains for *Scn1a*$^{+/+}$, n=18 cortical hemispheres over 9 independent brains for *Scn1a*$^{Stop/+}$, and n=10 cortical hemispheres over 5 independent brains for *Scn1a*$^{Stop/+}$ -Cre;** p=0.001; hippocampus, * p=0.0148, ** p=0.0074 n=6 brains for each genotype; cerebellum, ** p=0.0019, * p=0.0411, n=7 brains for each genotype; striatum, ** p=0.0054, * p=0.0288, n=9 brains for each genotype, Kruskal-Wallis followed by Dunn's multiple comparisons test). All data shown are means ± SEM, with dots representing individual samples. Source data are provided as a Source Data file.

Supplementary Figure 3

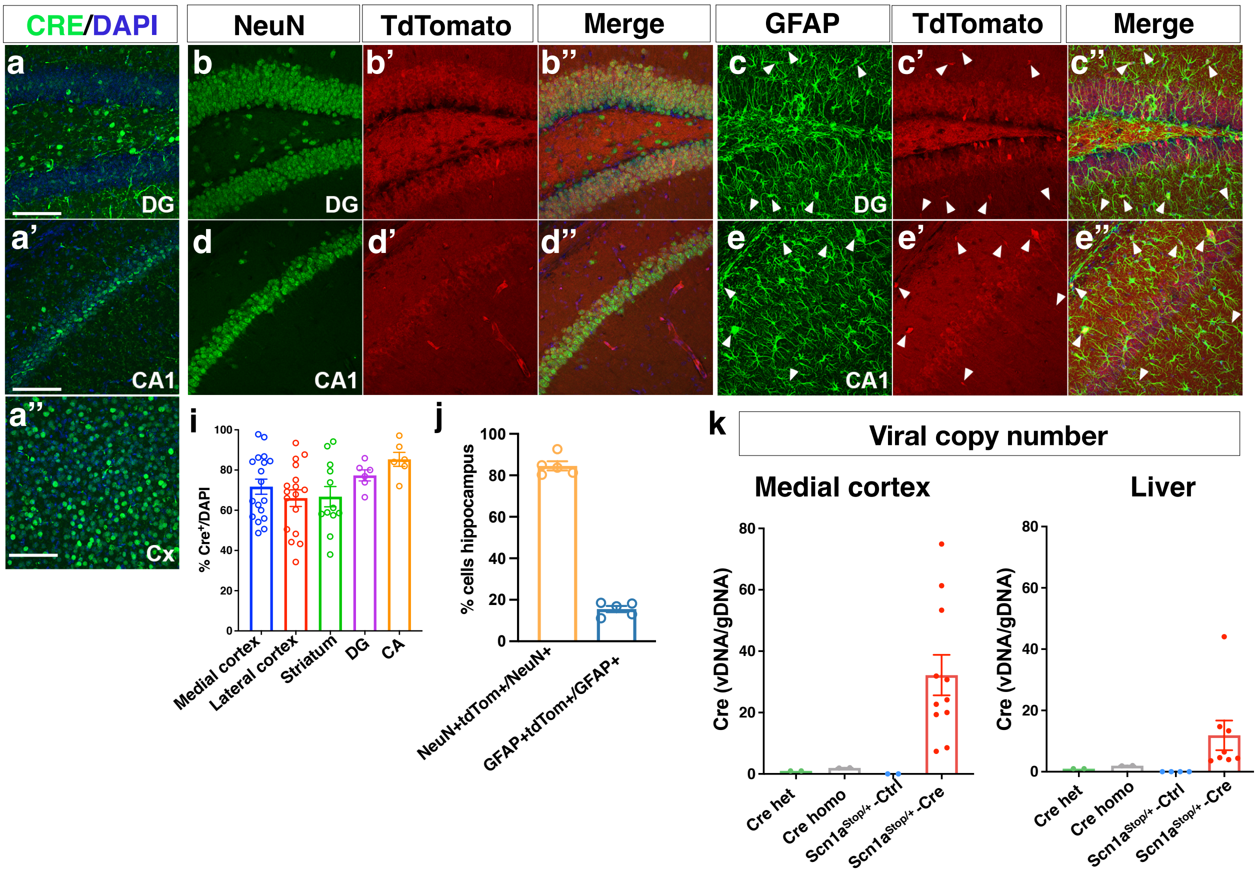

Supplementary Figure 3| PHP.eB-mediated Cre recombinase gene transfer in P30 *Scn1a*$^{Stop/+}$ mice.
(**a-a''**) Representative images in different areas of immunofluorescence for Cre protein on brain sections from *Scn1a*$^{Stop/+}$- Cre mice. DG, dentate girus, CA1, cornus ammonis, Cx, cortex; (**b-e''**) Representative sections of the hippocampus (DG and CA1) of PHP.eB-Cre injected Ai14 mice stained with NeuN and GFAP (**i**) Quantification of the percentage of Cre expressing cells on the total of Dapi for each brain area, n=3 independent brains, sections n=18 for medial and lateral Cx, n=12 for St, n=6 for DG and CA. (**j**) Quantification of the percentage of NeuN tdTomato double positive cells (transduced neurons) over the total of NeuN positive and the percentage of GFAP tdTomato double positive cells (transduced glial cells) over the total of GFAP positive n=2 independent brains, sections n=5 (**k**) Evaluation of the number of viral Cre copies in the cerebral cortex (n=11 cortical hemispheres, 6 independent mice) and liver (n=8 liver biopsies, 6 independent mice) of *Scn1a*$^{Stop/+}$- Cre injected mice. Viral copies of Cre were normalized on the endogenous *Lmn2b* gene and expressed relative to control heterozygous Cre knock-in mice. Data are shown as mean ± SEM. Scale bars, 100 µm. Source data are provided as a Source Data file.

Supplementary Figure 4

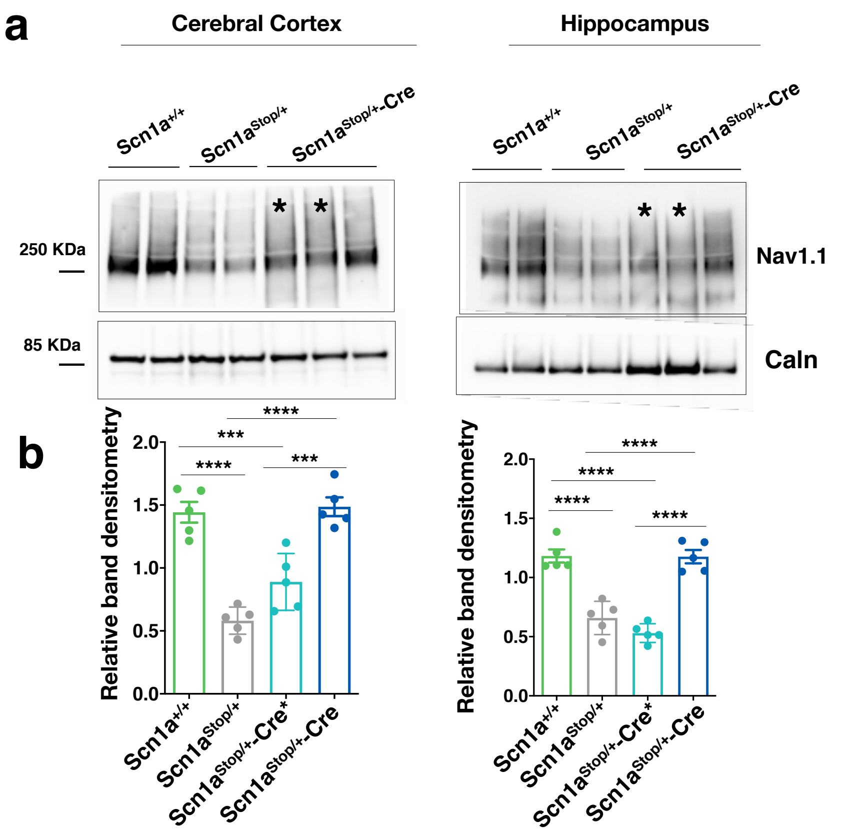

Supplementary Figure 4| Suboptimal expression of Na$_V$1.1 protein in *Scn1a$^{Stop/+}$* -Cre mice.
(**a**) Representative WB for Na$_V$1.1 and Calnexin protein obtained from cortical and hippocampal membrane-enriched protein extracts. Asterisks (*) indicates protein lysates of two mice in which Na$_V$ 1.1 protein level was not fully restored and died after thermo-induction. (**b**) Densitometric quantification of immunoreactive Na$_V$1.1 bands normalized on calnexin. All data shown are means ± SEM, with dots representing individual samples (****p<0.0001,***p=0.0007 for *Scn1a$^{+/+}$* vs *Scn1a$^{Stop/+}$*, ***p=0.0003 for *Scn1a$^{Stop/+}$*-Cre* vs *Scn1a$^{Stop/+}$*-Cre cortex, one-way ANOVA with Tukey's multiple comparison n=5 hemispheres, 4 independent brains for *Scn1a$^{+/+}$* and *Scn1a$^{Stop/+}$*, and n=4 hemispheres , 2 independent brains for *Scn1a$^{Stop/+}$* -Cre* and *Scn1a$^{Stop/+}$* -Cre). Data are shown as mean ± SEM. Source data are provided as a Source Data file.

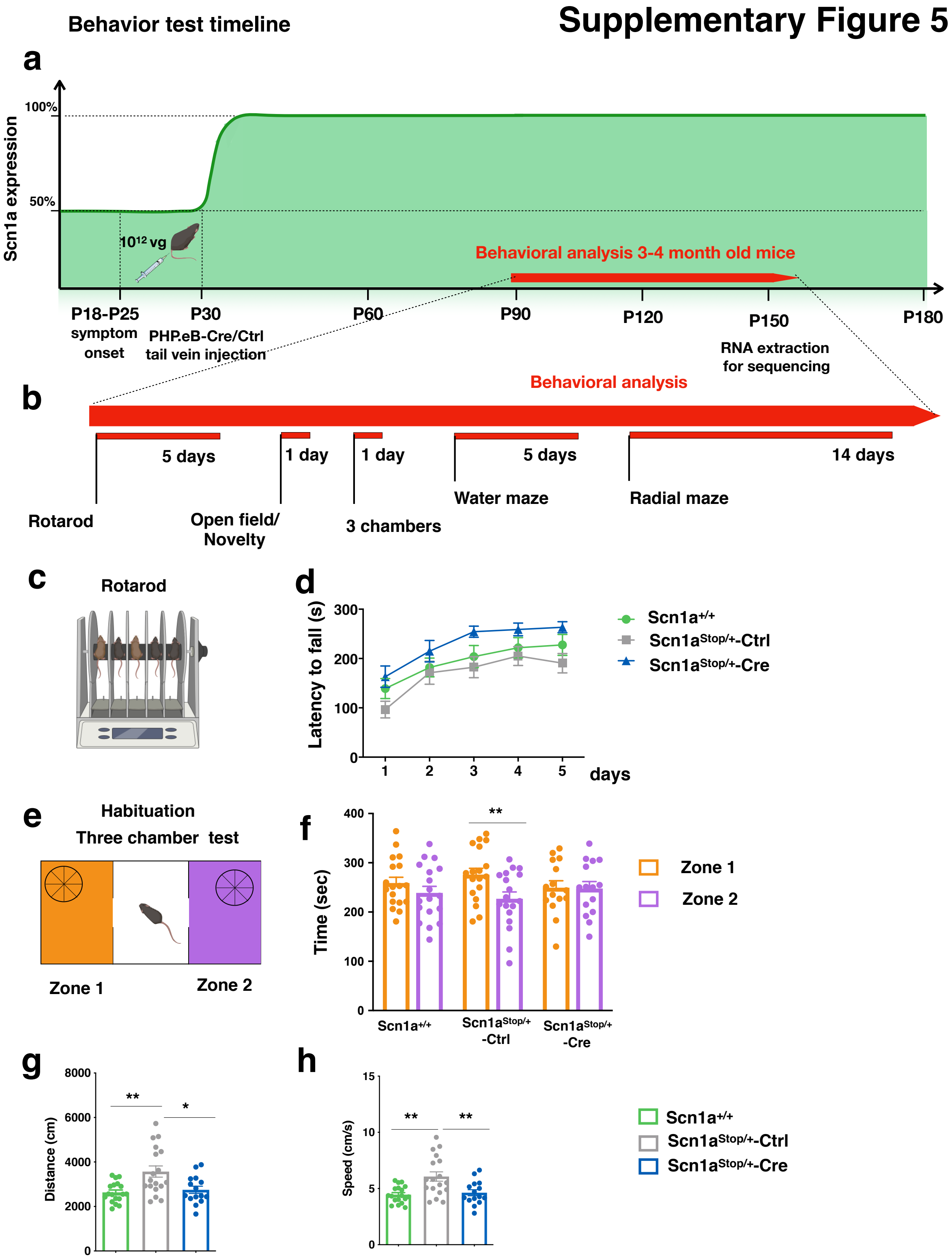

Supplementary Figure 5| Behavior test timeline

(**a, b**) Time-line of behavioral tests. (**c**) Scheme of the rotarod test; (**d**) Latency to fall from the from the accelerating rotarod, p=0.6098 two-way ANOVA, *Scn1a*$^{+/+}$ (n=17; *Scn1a*$^{Stop/+}$- Ctrl n=16 and *Scn1a*$^{Stop/+}$-Cre n = 14). (**e**) Scheme of the habituation in the three chamber test; (**f**) Time spent in Zone 1 and Zone 2 by the three groups of animals, *Scn1a*$^{+/+}$ n=18; *Scn1a*$^{Stop/+}$- Ctrl n=18 and *Scn1a*$^{Stop/+}$-Cre n = 15, (**p=0.009, two-way ANOVA, with Tukey's multiple comparison). (**g, h**) Distance and speed during the three chamber test, (*p=0.01, **p=0.0019 for g and **p=0.0013 and **p=0.0064 for h, one-way ANOVA, with Tukey's multiple comparison, *Scn1a*$^{+/+}$ n=18; *Scn1a*$^{Stop/+}$- Ctrl n=18 and *Scn1a*$^{Stop/+}$-Cre n = 15). Data are shown as mean ± SEM, with dots representing individual mice. Source data are provided as a Source Data file. a, c, e, were Created with BioRender.com.

Supplementary Figure 6

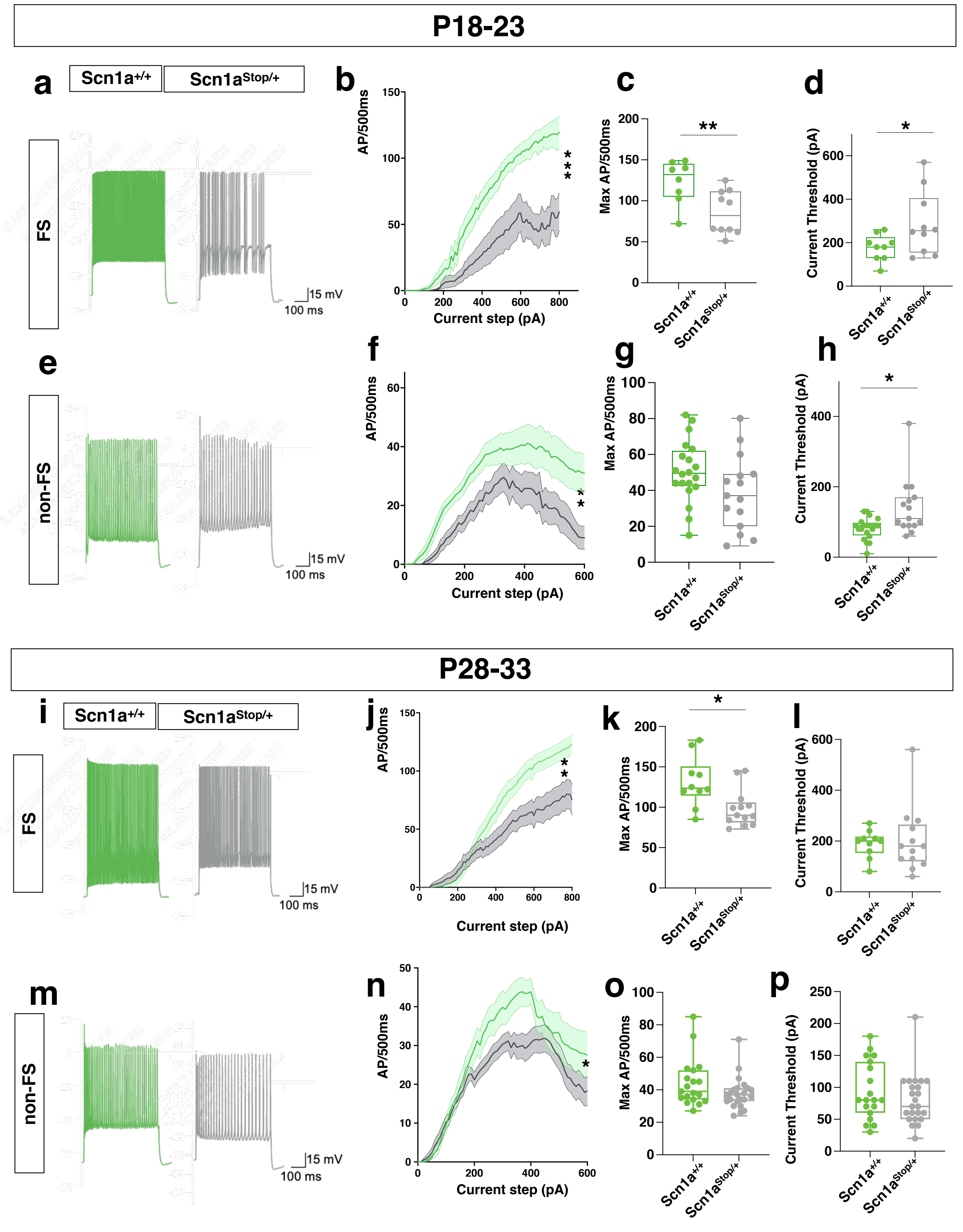

Supplementary Figure 6| Electrophysiological characterization of CA1 Gad67GFP+ interneurons from P18-P23 and P28-P33 mice. (**a**)Representative traces of FS interneurons in CA1 of *Scn1a*$^{+/+}$ and *Scn1a*$^{Stop/+}$ mice in response to 500 pA current step. (**b**) Input/output (I/O) curve for FS Gad67GFP+ interneurons from p18/23 mice (n=9 for *Scn1a*$^{+/+}$ from 6 animals, n=10 for *Scn1a*$^{Stop/+}$ -Ctrl from 4 animals ***p=0.0004, Two-way ANOVA); (**c**) Maximal AP number

in response to various intensity 500 ms current steps for FS (n=9 for *Scn1a*$^{+/+}$, n=10 for *Scn1a*$^{\text{Stop/+}}$, ** p=0.0094, unpaired two sided t-test), (**d**) Current threshold (n=9 for Scn1a$^{+/+}$ from 6 animals, n=10 for Scn1a$^{\text{Stop/+}}$ -Ctrl from 4 animals *p=0.0492 , unpaired two sided t-test). (**e**) Representative traces of non-FS interneurons in CA1 of *Scn1a*$^{+/+}$ and *Scn1a*$^{\text{Stop/+}}$ mice in response to 250 pA current step. **f**) Input/output curve for non-FS Gad67GFP+ interneurons (n=20 for *Scn1a*$^{+/+}$, n=15 for *Scn1a*$^{\text{Stop/+}}$ -Ctrl **p=0.0110, Two-way ANOVA with Bonferroni multiple comparison). (**k**) Maximal AP number in response to various intensity 500 ms current steps for non-FS (n=20 for Scn1a$^{+/+}$ from 6 animals, n=15 for Scn1a$^{\text{Stop/+}}$ -Ctrl from 4 animals p=0. 0804, unpaired two sided t-test), (**d**) Current threshold (n=20 for *Scn1a*$^{+/+}$ from 6 animals, n=15 for *Scn1a*$^{\text{Stop/+}}$ -Ctrl from 4 animals *p=0. 0150, unpaired two sided t-test). (**i**)Representative traces of FS interneurons in CA1 of *Scn1a*$^{+/+}$ and *Scn1a*$^{\text{Stop/+}}$ mice in response to 500 pA current step. (**j**) I/O curve for FS Gad67GFP+ interneurons from P28-33 mice (n=13 for *Scn1a*$^{+/+}$ from 7 animals , n=10 for $^{\mathit{Scn1a}\text{Stop/+}}$ -Ctrl from 6 animals **p=0.0154, Two-way ANOVA with Bonferroni multiple comparison); (**k**) Maximal AP number in response to various intensity 500 ms current steps for FS (n=13 for *Scn1a*$^{+/+}$ from 7 animals, n=10 for *Scn1a*$^{\text{Stop/+}}$ -Ctrl from 6 animals *p=0.0125 , unpaired two sided t-test), (**l**) Current threshold (n=9 for Scn1a$^{+/+}$ from 7 animals, n=10 for *Scn1a*$^{\text{Stop/+}}$ -Ctrl from 6 animals *p=0.0492 , unpaired two sided t-test). (**m**) Representative traces of non-FS interneurons in CA1 of *Scn1a*$^{+/+}$ and *Scn1a*$^{\text{Stop/+}}$ mice in response to 250 pA current step. (**n**) I/O curve for non-FS Gad67GFP+ interneurons (n=19 for *Scn1a*$^{+/+}$ from 7 animals, n=20 for *Scn1a*$^{\text{Stop/+}}$ -Ctrl from 6 animals *p=0.0471, Two-way ANOVA with Bonferroni post hoc multiple comparison). (**o**) Maximal AP number in response to various intensity 500 ms current steps, (**p**) Current threshold. Data shown are means ± SEM for b, f, j and n; for box plot in b, c, d, f, g, h, j, k, l, n, o and p each dot represent mean values from each cell, central lines median value and box limits represent 25% and 75% percentiles, while whiskers minimal and max values (data available in Extendend legend file) Source data are provided as a Source Data file.

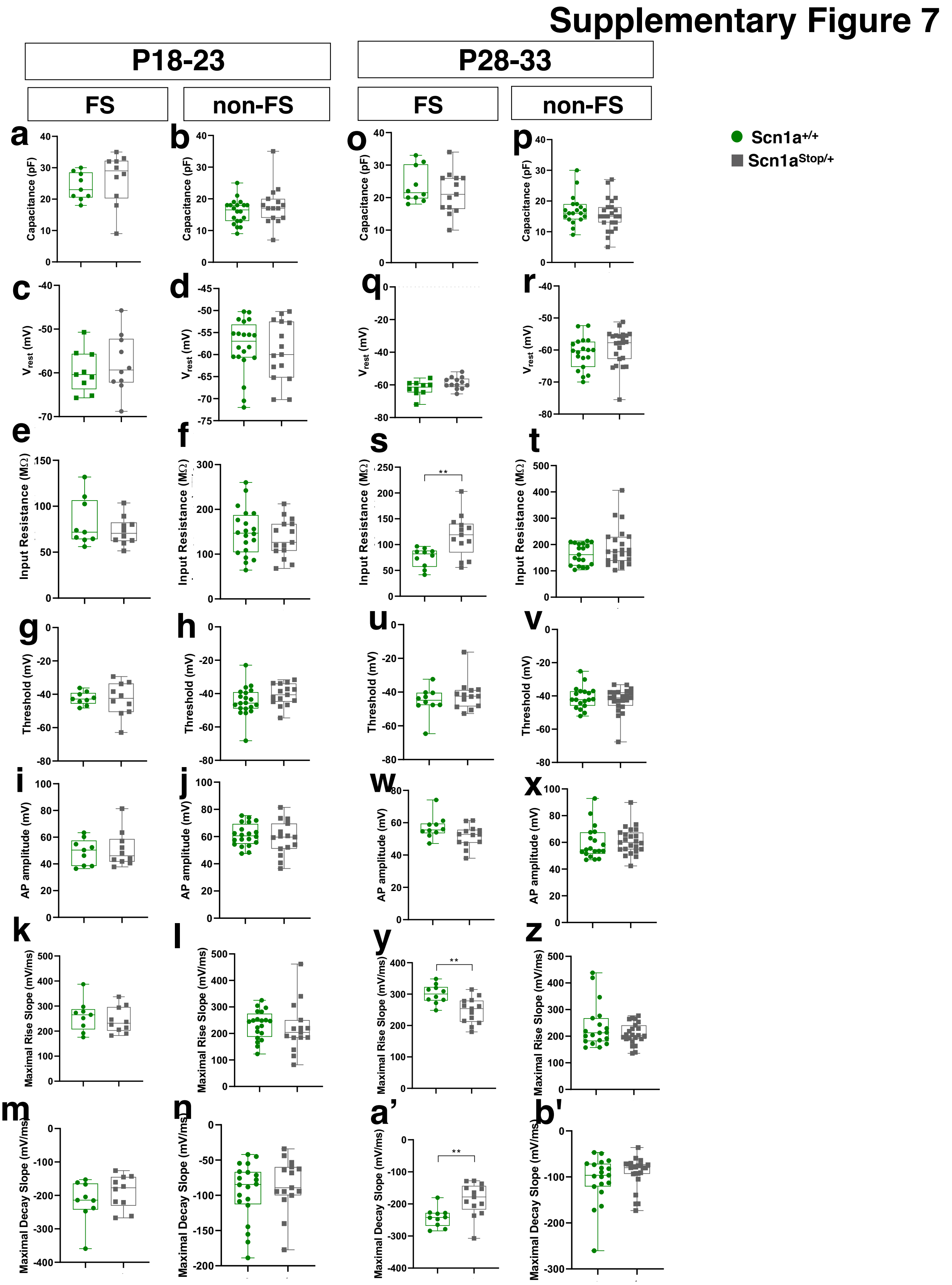

Supplementary Figure 7| Passive and active cell properties in GAD67-GFP cells at P18-23(a-n) and P28-33(o-b')
(**a**) Capacitance, (**c**) resting membrane potential, (**e**) input resistance, (**g**) action potential voltage threshold, (**i**) amplitude, (**k**) maximal rise slope, (**m**) maximal decay slope in FS CA1 Gad67GFP+ interneurons from P18/23 mice (n=9 for Scn1a$^{+/+}$ from 6 animals, n=10 for Scn1a$^{Stop/+}$ -Ctrl from 4 animals); (**b**) Capacitance, (**d**) resting membrane potential, (**f**) input resistance, (**h**) action potential voltage threshold, (**j**) amplitude, (**l**) maximal rise slope, (**n**) maximal decay slope

in non-FS CA1 Gad67GFP+ interneurons from P18/23 mice (n=20 for *Scn1a*$^{+/+}$, n=15 for *Scn1a*$^{Stop/+}$ -Ctrl).(**o**) Capacitance, (**q**) resting membrane potential, (**s**) input resistance (n=10 for *Scn1a*+/+ from 7 animals, n=13 for *Scn1a*Stop/+ -Ctrl ** from 6 animals p=0,0038, unpaired two sided t-test), (**u**) action potential voltage threshold, (**w**) amplitude, (**y**) maximal rise slope (n=10 for *Scn1a*$^{+/+}$ from 7 animals, n=13 for *Scn1a*Stop/+ -Ctrl from 6 animals **p=0,0018, unpaired two sided t-test), (**a'**) maximal decay slope (n=10 for *Scn1a*$^{+/+}$ from 7 animals, n=13 for *Scn1a*Stop/+ -Ctrl from 6 animals **p=0,0033, unpaired two sided t-test) in FS CA1 Gad67GFP+ interneurons from P28/33 mice; (**p**) Capacitance, (**r**) resting membrane potential, (**t**) input resistance, (**v**) action potential voltage threshold, (**x**) amplitude, (**z**) maximal rise slope, (**b'**) maximal decay slope in non-FS CA1 Gad67GFP+ interneurons from P28/33 mice (n=19 for *Scn1a*$^{+/+}$ from 7 animals, n=20 for *Scn1a*$^{Stop/+}$ -Ctrl from 6 animals). For box plot each dot represent mean values from each cell, central lines median value and box limits represent 25% and 75% percentiles, while whiskers minimal and max values (data available in Extendend legend file). Source data are provided as a Source Data file.

Supplementary Figure 8

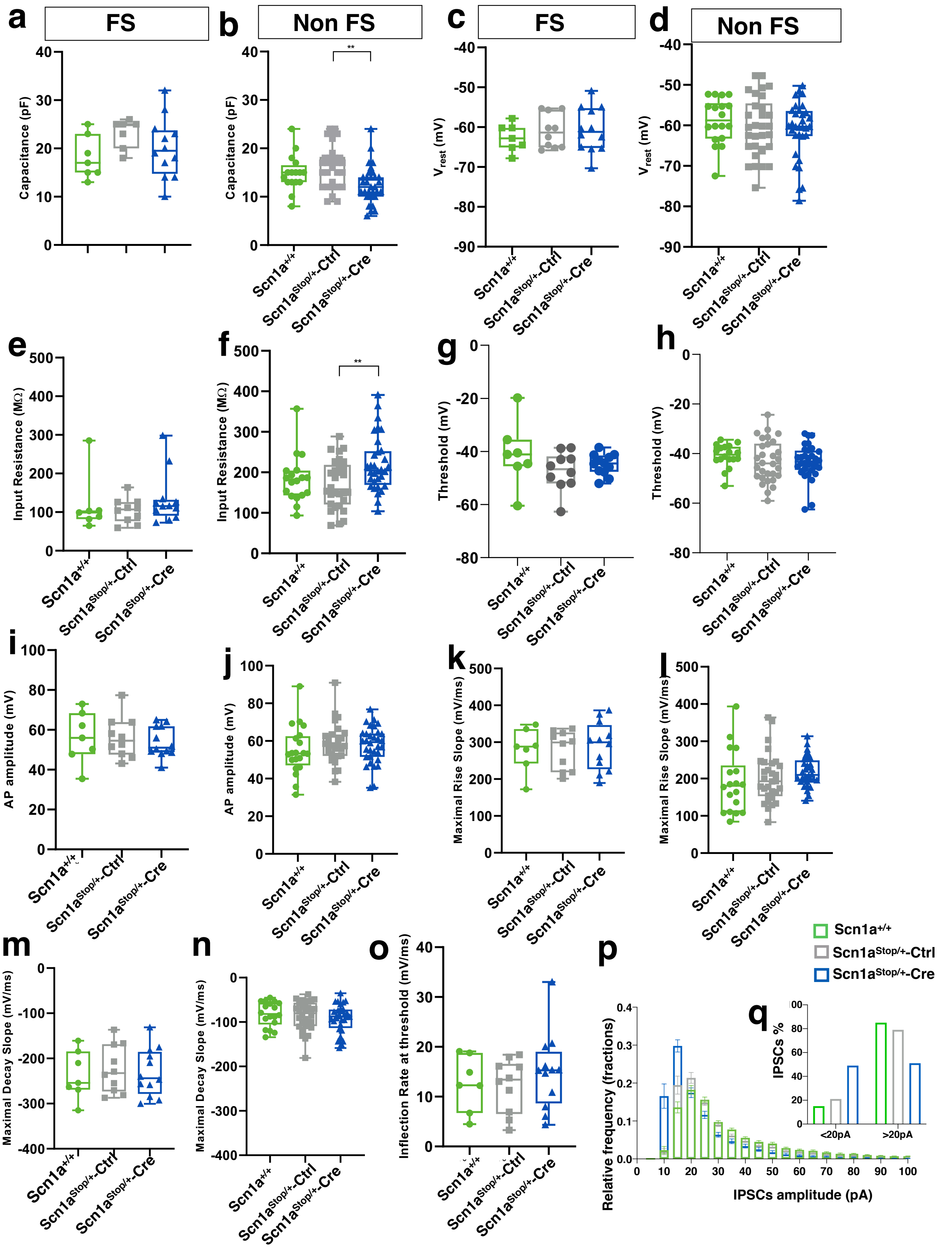

Supplementary Figure 8| Passive and active cell properties in GAD67-GFP cells at P45-55. (**a**) Cell capacitance, (**c**) Resting membrane potential, (**e**) input resistance, (**g**) action potential threshold, (**i**) amplitude, (**k**) maximal rise slope, (**m**) maximal decay slope and (**o**) inflection rate at threshold for FS CA1 interneurons (n=7 for *Scn1a*$^{+/+}$ from 4 animals,

n=10 for *Scn1a*$^{Stop/+}$ -Ctrl from 6 animals and n=12 for *Scn1a*$^{Stop/+}$-Cre from 5 animals). (**b,d,f, h, j, l, n**) The same properties in non-FS CA1 interneurons (n=18 for *Scn1a*$^{+/+}$ from 4 animals, n=30 from 6 animals for *Scn1a*$^{Stop/+}$ -Ctrl and n=32 from 5 animals for *Scn1a*$^{Stop/+}$-Cre. Cell capacitance and input resistance show alterations in *Scn1a*$^{Stop/+}$-Cre interneurons (One-way ANOVA with Tukey's post hoc comparison; **p=0.0014 and **p= 0.0075, respectively). (**m**) IPSCs amplitude distribution. (p<0.0001 Friedman ANOVA, with Dunn's post hoc multiple comparison) (n=28 for *Scn1a*$^{+/+}$ from 6 mice, n=21 for *Scn1a*$^{Stop/+}$ -Ctrl from 4 mice and n=26 for *Scn1a*$^{Stop/+}$-Cre from 4 mice). For box plot each dot represent mean values from each cell, central lines median value and box limits represent 25% and 75% percentiles, while whiskers minimal and max values (data available in Extendend legend file). Data shown are means ± SEM in p. Source data are provided as a Source Data file.

Supplementary Figure 9

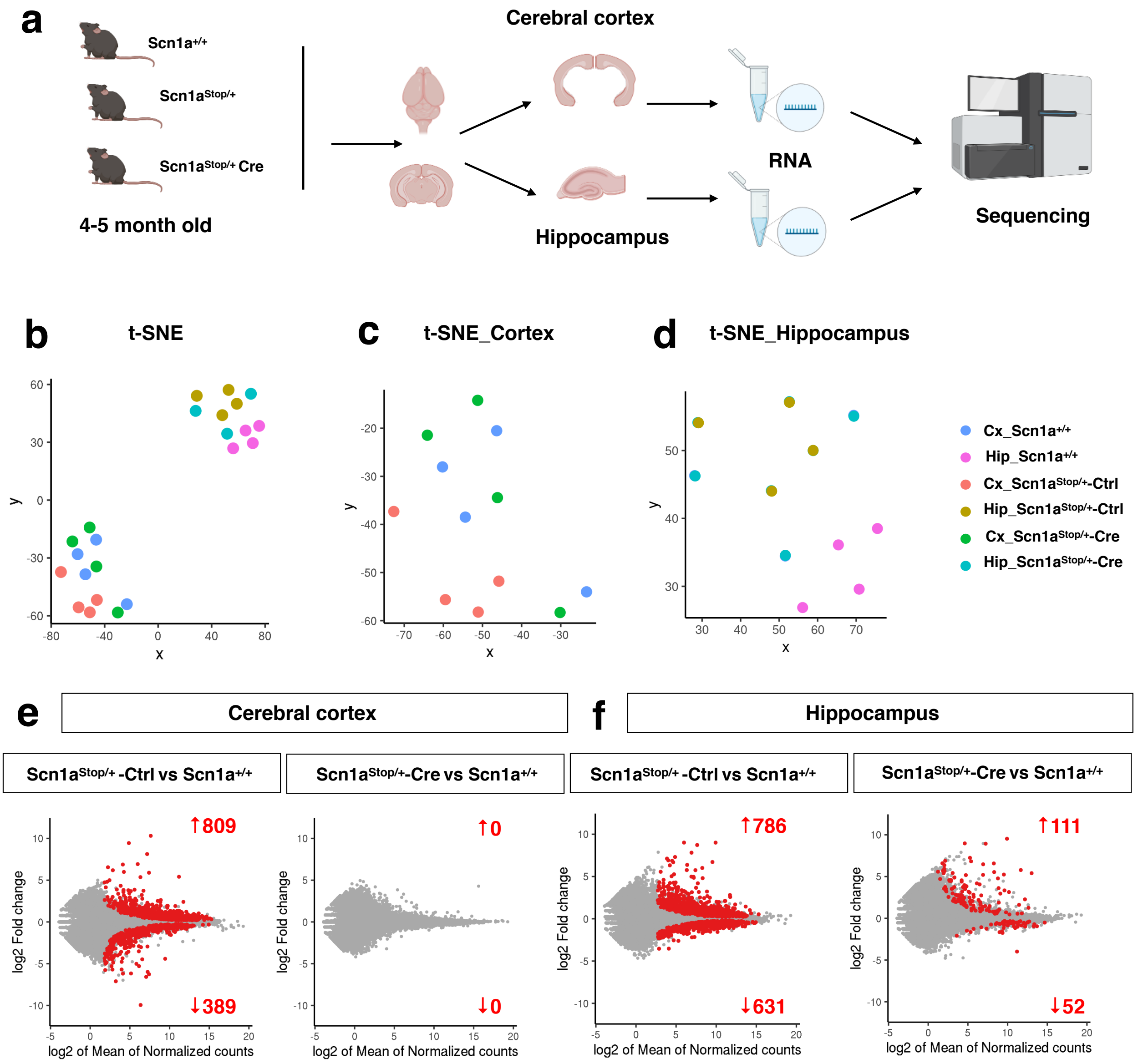

Supplementary Figure 9| Gene expression alterations characteristic of *Scn1a*$^{Stop/+}$-Ctrl are rescued in *Scn1a*$^{Stop/+}$-Cre mice (**a**) Experimental workflow for RNA-seq analyses on cerebral cortex and hippocampal tissue. Created with BioRender.com. (**b**) t-SNE analyses on the cortex and hippocampus of *Scn1a*$^{+/+}$, *Scn1a*$^{Stop/+}$-Ctrl and *Scn1a*$^{Stop/+}$-Cre

mice and gene clustering among relative tissue genotypes in (**c**) and (**d**). (**e, f**) Representation of DEGs in the cortex and in the hippocampus revealing several deregulated genes in *Scn1a*$^{Stop/+}$ -Ctrl mice compared to control mice and relative rescue in *Scn1a*$^{Stop/+}$-Cre mice.

Supplementary Figure 10

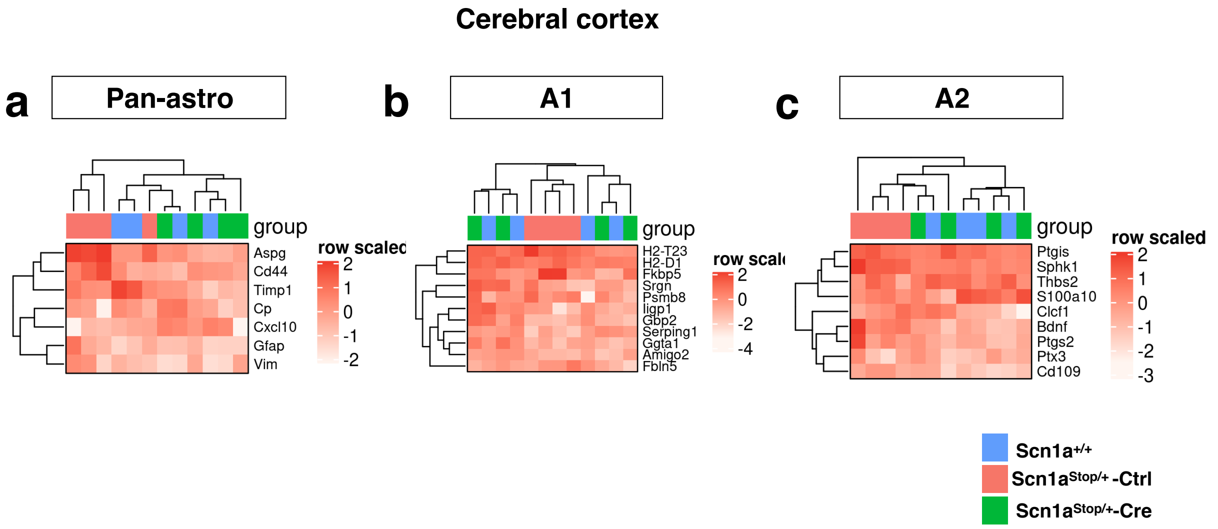

Supplementary Figure 10| Heatmaps showing the different level of expression of makers of pan-astrocytosis (**a**) A1 type reactive astrocytes (**b**), A2 type reactive astrocytes (**c**) in the cerebral cortex of the 3 experimental groups.

Supplementary Figure 11

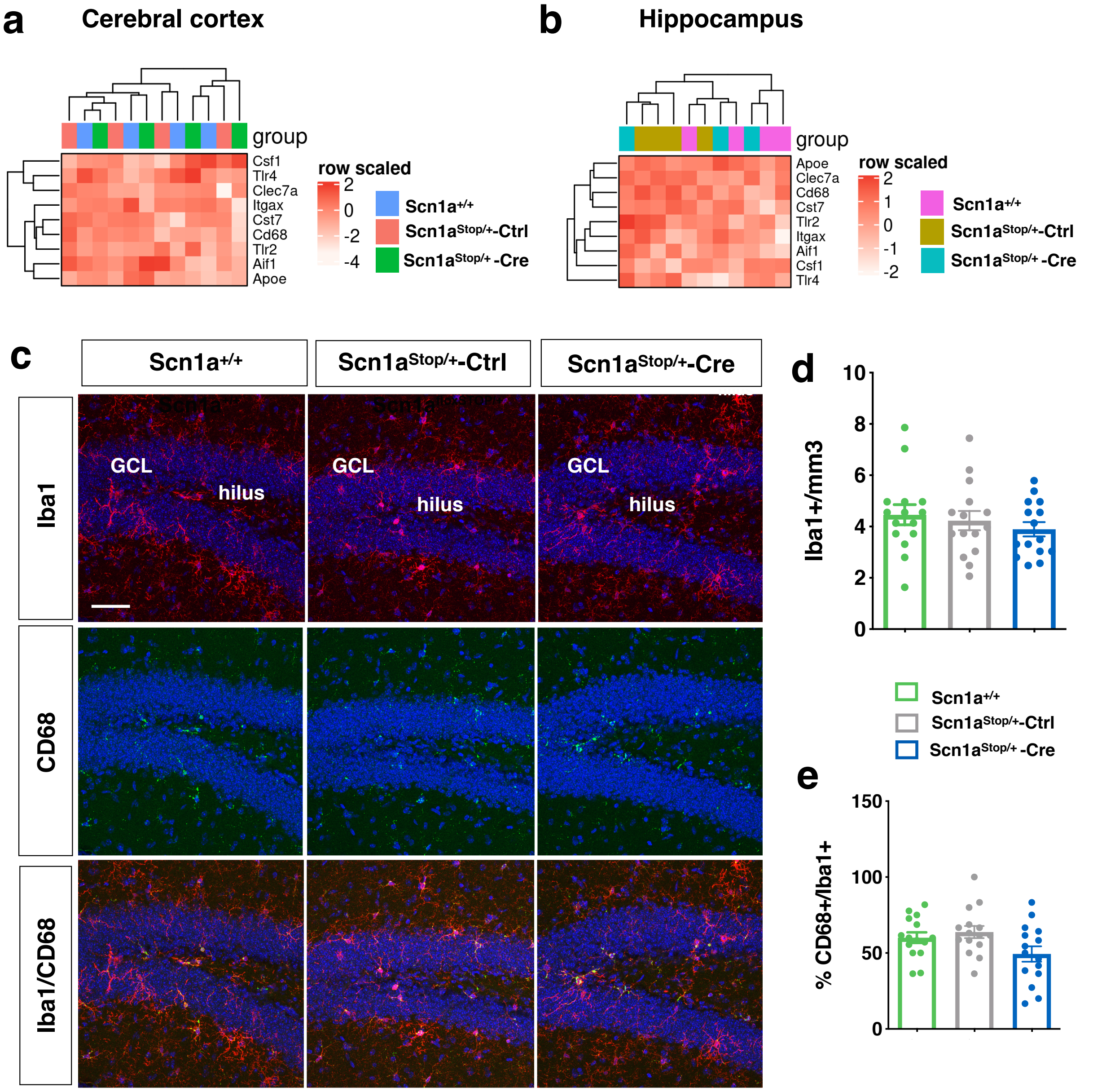

Supplementary Figure 11| Microglial marker analyses by RNA-seq and immunofluorescence staining
(**a,b**) Heatmaps showing activated microglial in the cortex nor in the hippocampus. (**c**) Immunofluorescence for Iba1 and CD68 in DG of 8-week-old *Scn1a*$^{+/+}$, *Scn1a*$^{Stop/+}$ -Ctrl and *Scn1a*$^{Stop/+}$- Cre mice, scale bar 100 µm (**d**) Quantification of Iba1+ cells and of CD68+/Iba1+ cells in the DG, n=15 images over 3 independent mice for genotype. Data are represented as means ± SEM. Source data are provided as a Source Data file.

Supplementary Figure 12

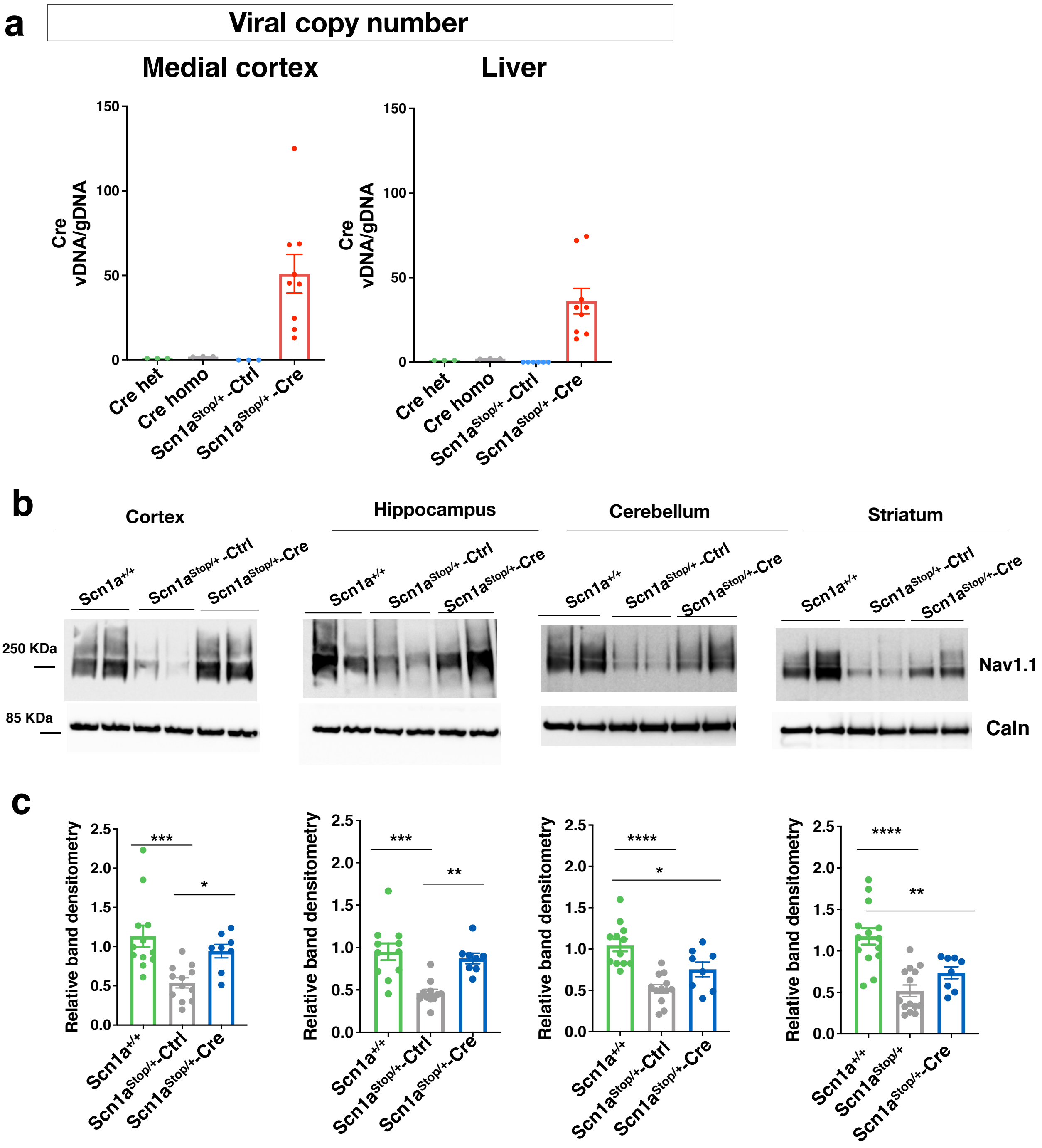

Supplementary Figure 12| PHP.eB-mediated Cre recombinase gene transfer in P90 *Scn1a*$^{Stop/+}$ mice. Evaluation of the number of viral Cre copies in the cerebral cortex and liver of *Scn1a*$^{Stop/+}$- Cre mice injected at P90. Viral copies of Cre were normalized on the endogenous *Lmn2b* gene and expressed relative to control heterozygous Cre knock-in mice (n=9 tissue biopsies over 6 independent mice). Data are shown as mean ± SEM, with dots representing individual mice. (**b**) Representative WB for Na$_V$1.1 and Calnexin protein obtained from membrane-enriched protein extracts. (**c**) Densitometric quantification of immunoreactive Na$_V$1.1 bands normalized on calnexin are presented (cerebral cortex, *** p=0.0009;* p=0.0127; hippocampus, *** p=0.0006, ** p=0.0061, Kruskal-Wallis followed by

Dunn's multiple comparisons test; cerebellum, **** p<0.0001, * p=0.0244; striatum,****p<0.0001, ** p=0.0066, one-way ANOVA with Tukey's multiple comparison). Data are shown as mean ± SEM; where present, dots represent individual samples. Source data are provided as a Source Data file.

Supplementary Data 1: DEGs

Differential gene expression analysis was performed with DESeq2 by Wald significance test and false discovery rate to adjust for multiple comparisons.

Supplementary Data 2: GO analysis of DEGs
