# Supplementary material for: Scn1a gene reactivation after symptom onset rescues pathological phenotypes in a mouse model of Dravet syndrome
Source: Nat Commun. 2022 Jan 10;13:161. doi: 10.1038/s41467-021-27837-w (PMC8748984; doi:10.1038/s41467-021-27837-w)
Supplement: Supplementary file 2 — Reporting Summary [file 41467_2021_27837_MOESM2_ESM.pdf]

nature portfolio

Corresponding author(s): Gaia Colasante and Vania Broccoli

Last updated by author(s): Nov 30 2021

Reporting Summary

Nature Portfolio wishes to improve the reproducibility of the work that we publish. This form provides structure for consistency and transparency in reporting. For further information on Nature Portfolio policies, see our Editorial Policies and the Editorial Policy Checklist.

Statistics

For all statistical analyses, confirm that the following items are present in the figure legend, table legend, main text, or Methods section.

n/a	Confirmed	
☐	☒	The exact sample size (*n*) for each experimental group/condition, given as a discrete number and unit of measurement
☐	☒	A statement on whether measurements were taken from distinct samples or whether the same sample was measured repeatedly
☐	☒	The statistical test(s) used AND whether they are one- or two-sided<br>*Only common tests should be described solely by name; describe more complex techniques in the Methods section.*
☐	☒	A description of all covariates tested
☐	☒	A description of any assumptions or corrections, such as tests of normality and adjustment for multiple comparisons
☐	☒	A full description of the statistical parameters including central tendency (e.g. means) or other basic estimates (e.g. regression coefficient) AND variation (e.g. standard deviation) or associated estimates of uncertainty (e.g. confidence intervals)
☐	☒	For null hypothesis testing, the test statistic (e.g. *F*, *t*, *r*) with confidence intervals, effect sizes, degrees of freedom and *P* value noted<br>*Give P values as exact values whenever suitable.*
☒	☐	For Bayesian analysis, information on the choice of priors and Markov chain Monte Carlo settings
☒	☐	For hierarchical and complex designs, identification of the appropriate level for tests and full reporting of outcomes
☒	☐	Estimates of effect sizes (e.g. Cohen's *d*, Pearson's *r*), indicating how they were calculated

Our web collection on statistics for biologists contains articles on many of the points above.

Software and code

Policy information about availability of computer code

Data collection	For EEG analysis, Neuroscore software version 3.3.931B-1 (DSI) was used; For behavioral tests Ethovision XT, Noldus was employed;Photoshop CC2015 and ImageJ were used for immunofluorescence analysis.
Data analysis	All statistical analysis was done using Prism 9 (GraphPad) and SPSS. For RNAseq, differential gene expression analysis was performed using DESeq2, STAR was employed for mapping the sequences on reference genome mm10 (https://www.gencodegenes.org/mouse/).

For manuscripts utilizing custom algorithms or software that are central to the research but not yet described in published literature, software must be made available to editors and reviewers. We strongly encourage code deposition in a community repository (e.g. GitHub). See the Nature Portfolio guidelines for submitting code & software for further information.

Data

Policy information about availability of data

All manuscripts must include a data availability statement. This statement should provide the following information, where applicable:

- Accession codes, unique identifiers, or web links for publicly available datasets
- A description of any restrictions on data availability
- For clinical datasets or third party data, please ensure that the statement adheres to our policy

Transcriptomic data are available in the NCBI Gene Expression Omnibus repository with the GSE171191 GEO ID (go to https://www.ncbi.nlm.nih.gov/geo/query/acc.cgi?acc=GSE171191). DEGs among different genotypes analyzed are provided in Supplementary Table 1 while GO analysis in Supplementary Table 2. Source data are provided.

Field-specific reporting

Please select the one below that is the best fit for your research. If you are not sure, read the appropriate sections before making your selection.

- [x] Life sciences
- [ ] Behavioural & social sciences
- [ ] Ecological, evolutionary & environmental sciences

For a reference copy of the document with all sections, see nature.com/documents/nr-reporting-summary-flat.pdf

Life sciences study design

All studies must disclose on these points even when the disclosure is negative.

Sample size	For EEG and behavioral analysis, no statistical methods were used to pre-determine sample sizes, but our sample sizes are similar to those reported in previous publications (doi.org/10.1111/epi.16482 and https://doi.org/10.1073/pnas.1906833116). For RNA seq, qPCR, WB and IHC sample size is indicated in Figure legends.
Data exclusions	One sample of the RNA_seq indicated as 19C in metadata was excluded as it was a cortical tissue but it profiled like an hippocampus, indicating a contamination during the dissection of the tissue.
Replication	All attempts at replication were successful. The number of independent replicates for each experiment is indicated in figure legends.
Randomization	For EEG analysis in Dravet mice injected at P30, animals were randomized after the assessment of spontaneous seizures during routine handling between the groups injected with PHP.eB Cre or Ctrl virus. For EEG analysis in Dravet mice injected at P90, animals were randomized after 2 weeks of EEG recordings of seizure baseline. For other experiments, randomization was not relevant as there was no selection of samples.
Blinding	Researchers involved in the experiments were blinded during experiment and data analysis. Another researcher replaced cards with genotypes and virus injected (PHP.eB-Cre or-Ctrl) on animal cages with others associating experimental groups with letters. At the end of the analysis the resercher was unblinded.

Reporting for specific materials, systems and methods

We require information from authors about some types of materials, experimental systems and methods used in many studies. Here, indicate whether each material, system or method listed is relevant to your study. If you are not sure if a list item applies to your research, read the appropriate section before selecting a response.

Materials & experimental systems

n/a	Involved in the study	
[ ]	[x]	Antibodies
[ ]	[x]	Eukaryotic cell lines
[x]	[ ]	Palaeontology and archaeology
[ ]	[x]	Animals and other organisms
[x]	[ ]	Human research participants
[x]	[ ]	Clinical data
[x]	[ ]	Dual use research of concern

Methods

n/a	Involved in the study	
[x]	[ ]	ChIP-seq
[x]	[ ]	Flow cytometry
[x]	[ ]	MRI-based neuroimaging

Antibodies

Antibodies used	The following antibodies were used in this work: for western blot, primary antibodies for Nav1.1 (rabbit, 1:200, Millipore) and Calnexin (rabbit, 1:2000, Sigma-Aldrich) were used and anti-rabbit HRP (1:5000, Dako) was used as secondary antibody; for immunofluorescence anti-Cre recombinase (mouse, 1:1000, Millipore), anti-NeuN (rabbit, 1:1000, Abcam), anti-GFAP (chicken, 1:1000, Abcam), anti-Glutamine synthetase GS (mouse, 1:1000, Millipore), anti-Iba1 (rabbit, 1:1000, Wako), anti-CD68 (rat, 1:1000, Abcam), anti-Vimentin (chicken, 1:1000, Abcam).
Validation	Nav1.1 (rabbit, cat. AB5204 Millipore)<br>Action potential initiation in neocortical inhibitory interneurons.Li, T; Tian, C; Scalmani, P; Frassoni, C; Mantegazza, M; Wang, Y; Yang, M; Wu, S; Shu, Y PLoS biology 12 e1001944 2014.<br>Colasante G, Lignani G 2. 2020 Jan 8;28(1):235-253. doi: 10.1016/j.ymthe.2019.08.018. Mol Ther. Epub 2019 Sep 3.<br>dCas9-Based Scn1a Gene Activation Restores Inhibitory Interneuron Excitability and Attenuates Seizures in Dravet Syndrome Mice<br><br>Calnexin (rabbit, 1:2000, Sigma-Aldrich)<br><br>anti-rabbit HRP (1:5000, Dako)<br>anti-Cre recombinase (mouse, 1:1000, Millipore)

anti-NeuN (rabbit, 1:1000, Abcam),
anti-GFAP (chicken, 1:1000, Abcam)
anti-Glutamine synthetase GS (mouse, clone GS6 Millipore)

anti-CD68 (cat. ab125212, Abcam).
Derbis M et al. Short antisense oligonucleotides alleviate the pleiotropic toxicity of RNA harboring expanded CGG repeats. Nat Commun 12:1265 (2021).

anti-IBA1 (cat. 019-197441, Wako) rabbit polyclonal
Rauch JN, 2020, Nature, 580(7803):381-385 DOI: 10.1038/s41586-020-2156-5

anti-Vimentin (cat. ab24525, Abcam).
Bucur O et al. Nanoscale imaging of clinical specimens using conventional and rapid-expansion pathology. Nat Protoc 15:1649-1672 (2020).

Eukaryotic cell lines

Policy information about cell lines

Cell line source(s)	E14 129Ola mouseES were a kind gift of Dr. Dario Bonanomi (Ospedale San Raffaele, Milan); HEK293T were purchased at ATCC, Cat# CRL-3216.
Authentication	Cell lines were authenticated by the suppliers.
Mycoplasma contamination	All the cell lines employed were negative for mycoplama contamination (PCR-test)
Commonly misidentified lines (See ICLAC register)	No commonly misidentified cell lines were used in the study

Animals and other organisms

Policy information about studies involving animals; ARRIVE guidelines recommended for reporting animal research

Laboratory animals	Scn1aStop/+ knock-in mice (Mus musculus) were maintained in a Sv129 background and crossed with CMV-Cre (C57BL/6N) to generate the Scn1aRec/+ mice, with C57BL/6J to generate F1 mice for the study, and with GAD67-GFP (C57BL/6N) mice for patch-clamp analysis. B6.Cg-Gt(ROSA)26Sortm14(CAG-tdTomato)Hze/J (Ai14) are C57Bl6/N. Both male and female mice were used for all the experiments, except for behavior and cosequently RNAseq were only males were employed. For seizures rescue experiments mice were injected at P0, or at P30 or at P90 and sacrified 4-5 months later. For beavior 3-4 month old mice were employed.
Wild animals	This study did not involve wild animals.
Field-collected samples	The study did not involve samples collected from the field.
Ethics oversight	All procedures were performed according to protocols approved by the internal IACUC and reported to the Italian Ministry of Health according to the European Communities Council Directive 2010/63/EU.

Note that full information on the approval of the study protocol must also be provided in the manuscript.
