# Supplementary material for: Scn1a gene reactivation after symptom onset rescues pathological phenotypes in a mouse model of Dravet syndrome
Source: Nat Commun. 2022 Jan 10;13:161. doi: 10.1038/s41467-021-27837-w (PMC8748984; doi:10.1038/s41467-021-27837-w)
Supplement: Supplementary file 4 — Description of Additional Supplementary Files [file 41467_2021_27837_MOESM4_ESM.pdf]

Title: Supplementary Data 1:
Description: This file contains DEGs of RNAseq analysis 7

Title: Supplementary Data 2:
Description: This file contains GO of RNAseq analysis
